# Repeated Clozapine Increases the Level of Serotonin 5-HT_1A_R Heterodimerization with 5-HT_2A_ or Dopamine D_2_ Receptors in the Mouse Cortex

**DOI:** 10.3389/fnmol.2018.00040

**Published:** 2018-02-15

**Authors:** Marta Szlachta, Maciej Kuśmider, Paulina Pabian, Joanna Solich, Magdalena Kolasa, Dariusz Żurawek, Marta Dziedzicka-Wasylewska, Agata Faron-Górecka

**Affiliations:** Department of Pharmacology, Institute of Pharmacology, Polish Academy of Sciences, Kraków, Poland

**Keywords:** autoradiography, clozapine, haloperidol, heterodimers, ketamine, proximity ligation assay

## Abstract

G-protein–coupled receptor (GPCR) heterodimers are new targets for the treatment of schizophrenia. Dopamine D_2_ receptors and serotonin 5-HT_1A_ and 5-HT_2A_ receptors play an important role in neurotransmission and have been implicated in many human psychiatric disorders, including schizophrenia. Therefore, in this study, we investigated whether antipsychotic drugs (clozapine (CLZ) and haloperidol (HAL)) affected the formation of heterodimers of D_2_–5-HT_1A_ receptors as well as 5-HT_1A_–5-HT_2A_ receptors. Proximity ligation assay (PLA) was used to accurately visualize, for the first time, GPCR heterodimers both at *in vitro* and *ex vivo* levels. In line with our previous behavioral studies, we used ketamine to induce cognitive deficits in mice. Our study confirmed the co-localization of D_2_/5-HT_1A_ and 5-HT_1A_/5-HT_2A_ receptors in the mouse cortex. Low-dose CLZ (0.3 mg/kg) administered repeatedly, but not CLZ at 1 mg/kg, increased the level of D_2_–5-HT_1A_ and 5-HT_1A_–5-HT_2A_ heterodimers in the mouse prefrontal and frontal cortex. On the other hand, HAL decreased the level of GPCR heterodimers. Ketamine affected the formation of 5-HT_1A_–5-HT_2A_, but not D_2_–5-HT_1A_, heterodimers.

## Introduction

It is well established that dopamine D_2_ receptors and serotonin 5-HT_1A_ and 5-HT_2A_ receptors play an important role in neurotransmission and that alterations in their functioning are implicated in many human neurological and psychiatric disorders, including schizophrenia. For a long time, prominent focus has been placed on the antipsychotic effects of D_2_ receptor blockade (in the extended striatum), which alleviates the positive symptoms of schizophrenia. Only since the 1990s has there emerged an appreciation of the effects of atypical antipsychotic medications, which can be beneficial for the negative (cognitive) symptoms via combined action at dopamine as well as serotonin and other classes of neuroreceptors (Newman-Tancredi and Kleven, 2011). Considerable evidence has indicated that it is actually the balance between the properties of D_2_ receptors and 5-HT_1A_ receptors that has a profound influence on the profile of action of these drugs in preclinical models (Newman-Tancredi, 2010); however, these actions are also influenced by additional interactions at other receptor subtypes, such as 5-HT_2A_/5-HT_2C_, metabotropic glutamate receptor_2/3_ (mGluR_2/3_), and *N*-methyl-d-aspartate (NMDA) receptor (Gaur et al., 2008; Meltzer and Huang, 2008; Meltzer and Massey, 2011; Herrick-Davis, 2013; Łukasiewicz et al., 2016).

Clozapine (CLZ), an antipsychotic drug currently used in the clinic but still being the object of basic studies designed to search for its unique molecular features, has been also shown to act via various heterodimers. However, these studies have used *in vitro* systems (cell lines transfected with recombinant receptors) and sophisticated technology based on fluorescence resonance energy transfer. It has been shown that CLZ uncouples dopamine D_1_–D_2_ receptor heterodimers (Faron-Górecka et al., 2008) and that the effect is dependent on the concentration of CLZ as well as on the incubation time. Łukasiewicz et al. (2011) demonstrated the influence of CLZ and haloperidol (HAL) as well as other antipsychotic compounds on D_2_–5-HT_2A_ dimerization and on alterations of the pharmacological properties of D_2_–5-HT_2A_ heterodimers. Moreover, the affinity of D_1_ and 5-HT_2A_ receptors for CLZ depends on whether they are present in the plasma membrane separately or together with the D_2_ receptor (Faron-Górecka et al., 2008; Łukasiewicz et al., 2011). In another *in vitro* study, the D_2_ receptor was able to form constitutive heterodimers with another serotonin receptor, 5-HT_1A_, and CLZ significantly increased this interaction (Łukasiewicz et al., 2016). Therefore, in the present study, we investigated whether CLZ, administered to mice acutely or repeatedly in two doses, affected the formation of heterodimers of dopamine D_2_–5-HT_1A_ receptors as well as 5-HT_1A_–5-HT_2A_ receptors. For development the cognitive deficit we used ketamine (KET) as tool substance, according to our previous publication which showed that CLZ dose-dependently reversed KET-induced cognitive deficits (Szlachta et al., 2017). The method of choice to study endogenous G-protein–coupled receptor (GPCR) interaction in the native tissue is the *in situ* proximity ligation assay (PLA; Trifilieff et al., 2011; Perreault et al., 2016; Borroto-Escuela et al., 2017b; Szafran-Pilch et al., 2017). PLA was used to accurately visualize the heterodimers both *in vitro* and in *ex vivo* mouse brain.

## Materials and Methods

Our study was approved by the Bioethical Committee II at the Institute of Pharmacology, Polish Academy of Sciences, Kraków, Smetna 12, Poland (Number of Bioethical approval: #1196). The experiments were carried out in accordance with the Bioethical Committee.

### Animals

Male C57Bl/6J mice (approximately 26 g and 11 weeks of age) were purchased from Charles River, Germany. Mice were housed in a room with a 12-h light-dark cycle (lights on at 07:30), constant temperature (21 ± 2°C), and humidity (40%–50%) conditions in standard laboratory cages. Mice were housed 5 per cage with mild food deprivation (2.9 g of food pellets per day) and *ad libitum* access to water. Food deprivation and housing schedule were based on our previous behavioral experiments (Szlachta et al., 2017). However, mice used in biochemical studies were not included in behavior experiments (they constituted a separate group of animals).

### Chemicals

KET (10% aqueous solution of 115.34 mg/mL, Biowet, Poland) was dissolved in saline (SAL) to a concentration of 100 mg/kg. CLZ (Tocris, UK) was dissolved in 1M hydrochloric acid and then diluted in SAL. NaOH solution was added to buffer the solution to ~pH 6.5–7.0. HAL (SAL solution of 5 mg/kg, Polfa Warszawa S.A, Poland) was diluted in SAL. Experiments were carried out using two different paradigms: acute and sub-chronic administration. For the acute paradigm, drugs (SAL, KET and/or CLZ at 0.3 mg/kg; intraperitoneally [i.p.]) were administered twice (second administration was 24 h after the first one) and mice were decapitated 1 h after the second injection. This experimental paradigm resulted from the scheme of our previous behavioral studies (Szlachta et al., 2017). For the sub-chronic paradigm, drugs (KET or SAL) were repeatedly administered (i.p.) for seven consecutive days, followed by replacement with CLZ (0.3 or 1 mg/kg; i.p.) or HAL (0.1 mg/kg; i.p.) for the next 7 days. All injections were done once per day during this period. Mice were sacrificed 24 h after the last drug administration.

### Tissue Preparation

The brains were isolated from decapitated animals and ra pidly frozen on dry ice. Coronal brain sections (5 μm thicknes for PLA technique and 12 μm thickness for autoradiography experimemnts) were cut using a Jung CM 3000 cryostat microtome (Leica, Germany) by a standard procedure. The slices were thaw-mounted on gelatin-covered microscope slides, air-dried, and stored at −20°C until use. Brain sections were identified according to The Mouse Brain Atlas (Paxinos and Franklin, 2001). Coronal slides were taken approximately at 2.68 mm Bregma and 0.98 mm Bregma.

### [^3^H]Domperidone Binding to Dopamine D_2_ Receptors and Analysis of Autoradiograms

Tissue sections were pre-incubated in phosphate buffer (pH 7.4) at room temperature for 15 min to rehydrate the tissue sections and to remove potential endogenous dopamine. Brain slices were incubated for 90 min at room temperature in phosphate buffer (pH 7.4) with addition of 10 mM MgCL_2_ and 150 mM KCl containing 0.4 nM tritium-labeled domperidone ([^3^H]domperidone). [^3^H]domperidone concentration refers to the dissociation constant (Kd value) for the [^3^H]domperidone-dopamine D_2_ receptor (Knable and Weinberger, 1997; Krystal et al., 1999; Seeman et al., 2003; Żurawek et al., 2013). To determine non-specific binding, parallel tissue sections from the same animals were incubated in the same buffer as described above, enriched with 10 μM (+) butaclamol for 90 min at room temperature. After incubation, tissue sections were washed three times in ice-cold phosphate buffer (pH 7.4) for 10 min and once in ice-cold distilled water for 1 min. The sections were dried overnight under a gentle stream of air. The radiolabeled brain slices were loaded into a FujiFilm BAS Cassette and placed against a Fuji Imaging Plate (Fujifilm, Japan) with autoradiographic microscales (GE Healthcare) for 7 days. The obtained autoradiograms were analyzed and quantified using ImageGauge software (Fujifilm, Japan). The specific binding of radioligand to D_2_ receptor was calculated by subtracting non-specific binding images in adjacent brain slices from the total binding signal.

### [^3^H]8-OH-DPAT Binding to 5-HT_1A_ Receptors and Analysis of Autoradiograms

Brain slices were pre-incubated in 50 mM Tris–HCl buffer (pH 7.4) enriched with 120 mM NaCl, 4 mM CaCl_2_, and 0.01% ascorbic acid at room temperature for 15 min to rehydrate the tissue sections and to remove potential endogenous serotonin. Incubation buffer was the same as pre-incubation buffer but contained additionally 2 nM [^3^H]8-OH-DPAT. The tissue sections were incubated in that buffer for 60 min at room temperature to determine total binding. [^3^H]8-OH-DPAT concentration corresponded to the Kd value (Schiller et al., 2003). Parallel sections treated with 10 μM serotonin were incubated in the incubation buffer with 2 nM [^3^H]8-OH-DPAT to determine non-specific binding for 60 min at room temperature. Incubation was terminated by washing the slices twice in ice-cold 50 mM Tris–HCl (pH 7.4) enriched with 120 mM NaCl, 4 mM CaCl_2_, and 0.01% ascorbic acid for 10 min and once in ice-cold distilled water for 1 min. The sections were dried overnight under a gentle stream of air. Imaging and analysis of autoradiograms were carried out in the same way as in the case of [^3^H]domperidone binding to D_2_ receptors described above.

### [^3^H]Ketanserin Binding to 5-HT_2A_ Receptors and Analysis of Autoradiograms

To remove endogenous serotonin and to rehydrate the tissue sections, brain sections were pre-incubated in 50 mM Tris–HCl buffer (pH 7.4) enriched with 120 mM NaCl and 4 mM CaCl_2_ for 15 min at room temperature. To determine total binding, slides were incubated in the pre-incubation buffer supplemented with 2 nM [^3^H]ketanserin for 60 min at room temperature. [^3^H]ketanserin concentration refers to the Kd value for the [^3^H]ketanserin-serotonin 5-HT_2A_ receptor binding (Schiller et al., 2003). The buffer used in the incubation step was enriched with 10 μM mianserin to define non-specific binding and parallel brain sections were incubated in that buffer for 60 min at room temperature. Tissue sections were washed twice in ice-cold 50 mM Tris–HCl buffer (pH 7.4) for 10 min and once in ice-cold distilled water for 1 min, and then dried overnight under a gentle stream of air. Imaging and analysis of autoradiograms were carried out in the same way as described above.

### Immunohistochemistry on Paraffin-Embedded Tissue Sections

To co-localize D_2_/5-HT_1A_ receptors and 5-HT_1A_/5-HT_2A_ receptors in the brain, mouse brain was fixed in 4% formaldehyde for overnight, then embedded in paraffin blocks and sectioned using a microtome. To stain D_2_, 5-HT_1A_, and 5-HT_2A_ receptors, anti-D_2_ receptor antibody (H-50, sc-9113, Santa Cruz Biotechnology, Inc.; 1:50 dilution), anti-SR-1A antibody (C-19, sc-1459, Santa Cruz Biotechnology, Inc.; 1:50 dilution), and anti-5-HT_2A_ receptor antibody (ab66049, Abcam; 1:200 dilution) were used, respectively. Dilutions of secondary antibodies (Alexa Fluor 488 Donkey anti-Rabbit IgG, ref. A21206; Alexa Fluor 555 Donkey anti-Goat IgG, ref.A21432; Alexa Fluor 555 Donkey anti-Rabbit IgG, ref. A31572; Invitrogen) were prepared in 5% NDS in concentration 1:100 in all cases. Imaging was done by fluorescence microscope (Zeiss Axio Imager. A2, Poland) using a 20× objective and the following excitation wavelengths: 563 nm for 5-HT_1A_ receptor, 490 nm for D_2_R and 5-HT_2A_ receptor, and 358 nm for DAPI.

### Proximity Ligation Assay (PLA)

To determine receptor interaction in native tissue, PLA was used (Söderberg et al., 2008; Borroto-Escuela et al., 2013, 2014; Perreault et al., 2016). The method uses the proximity probes, composed of oligonucleotide-conjugated secondary antibodies, to recognize specific targets. Close proximity of targets allow for binding of probes, their hybridization, and subsequent formation of a circular DNA strand. Addition of polymerase in the next step leads to amplification of these DNA circles via polymerase chain reaction. The signal from each detected pair of proteins is visualized as an individual fluorescent spot.

PLA was performed according to the manufacturer’s protocol of the Duolink *in situ* PLA Probes and Duolink *in situ* Detection Reagents Orange kit (Cat. No. DUO92007 and Cat. No. DUO92008, respectively). According to Szafran-Pilch et al. (2017), brain sections of 5 μm thickness were used in PLA method. Antibodies, the same as used in immunohistochemistry, were dissolved in Antibody Diluent in concentration 1:100. For imaging, the slides were dried and mounted with a cover slip using ~7 μl Duolink *in situ* Mounting Medium with DAPI. Imaging and analysis of labeled brain sections was done with a fluorescence microscope (Zeiss Axio Imager. A2, Poland) using a 40× objective and excitation wavelength 563 nm for PLA and 358 nm for DAPI. From each coronal brain sections, four measurements in the prefrontal cortex and the frontal cortex were obtained. Each image was analyzed using microscope software to count individual fluorescent spots. Calculating a number of fluorescent spots was based on the measurement of intensity fluorescence level. Threshold for fluorescence intensity measurement was set manually, but calculations were done automatically by the software.

### Statistical Analysis

Raw data were analyzed using two-way analysis of variance (ANOVA) with Bonferroni post-test to compare all groups of the experiments. Results from the specific binding of all three radioligands as well as results from the PLA assay were normalized to the data of the control group (expressed as 100%).

## Results

### [^3^H]Domperidone Binding to Dopamine D_2_ Receptors and Analysis of Autoradiograms

**Figure 1:**
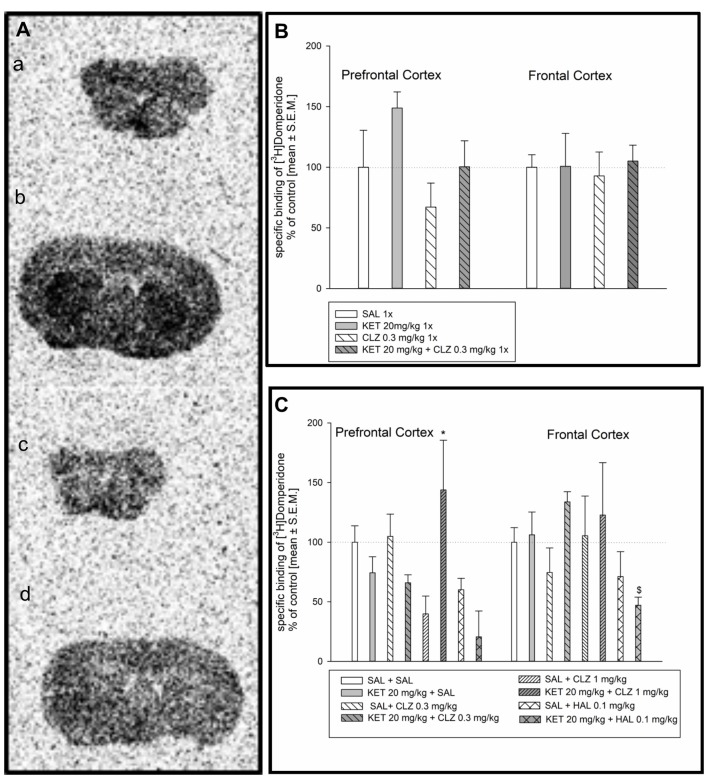
Effect of clozapine (CLZ) and haloperidol (HAL) treatment on specific binding of [^3^H]domperidone to dopamine D_2_R. **(A)** Representative autoradiograms of the total [^3^H]domperidone binding in mouse prefrontal cortex **(a)**; non-specific binding of [^3^H]domperidone in prefrontal cortex **(b)**; total [^3^H]domperidone binding in mouse frontal cortex **(c)**; non-specific binding of [^3^H]domperidone in frontal cortex **(d)**. **(B)** Acute administration of CLZ 0.3 mg/kg did not affect on density D_2_ receptor level in prefrontal and frontal cortex. **(C)** For repeated treatment, two-way analysis of variance (ANOVA; SAL, KET) × (SAL, CLZ 0.3 mg/kg, CLZ 1 mg/kg) revealed a significant interaction between KET and CLZ (*F*_(2,23)_ = 7.139; *p* < 0.01) as well as statistical analysis (SAL, KET) × (SAL, HAL) indicated a significant effects caused by HAL (*F*_(1,14)_ = 14.73; *p* < 0.05) and KET (*F*_(1,14)_ = 6.351; *p* < 0.01) on [^3^H]domperidone binding to D_2_ receptors in the prefrontal cortex. In the frontal cortex, using two-way ANOVA no significant effect was observed after sub-chronic drug’s treatment. The *post hoc* analysis revelead the statistical significant differences between: CLZ 1 mg/kg and KET + CLZ 1 mg/kg groups, *p < 0.05 and between KET 20 mg/kg and KET+HAL 0.1 mg/kg groups, ^$^*p* < 0.05. The control data were standardized to 100%. The data represent the mean ± SEM.

Radioligand binding determination to dopamine D_2_ as well as serotonin receptors (5HT_1A_ and 5HT_2A_) using autoradiography was done before PLA experiments to exclude possibility that changes in receptors dimerization level resulted from drug influence on receptors density. Representative autoradiograms of the distribution of [^3^H]domperidone-binding sites in the prefrontal cortex and frontal cortex of the mouse brain are presented in Figure 1A. Analysis of [^3^H]domperidone binding to dopamine D_2_ receptors in prefrontal cortex and frontal cortex in mouse brain did not show any significant differences after acute treatment of CLZ in dose 0.3 mg/kg (Figure 1B). In acute administration paradigm, only one dose of CLZ was used in line with our previous behavioral studies (Szlachta et al., 2017). Sub-chronic treatment of mice revealed increase in specific [^3^H]domperidone-binding sites in prefrontal cortex after treatment KET + CLZ 1 mg/kg in comparison to mice group treated SAL + CLZ 1 mg/kg (Figure 1C). However, KET + HAL caused decrease in D_2_ receptors density in relation to the KET + SAL group in the prefrotal cortex. Analysis of radioligand binding to dopamine D_2_ receptor in frontal cortex did not show any significant differences after treatment of all using antipsychotic drugs (Figure 1C).

### [^3^H]8-OH-DPAT Binding to Serotonin 5-HT_1A_ and Analysis of Autoradiograms

**Figure 2:**
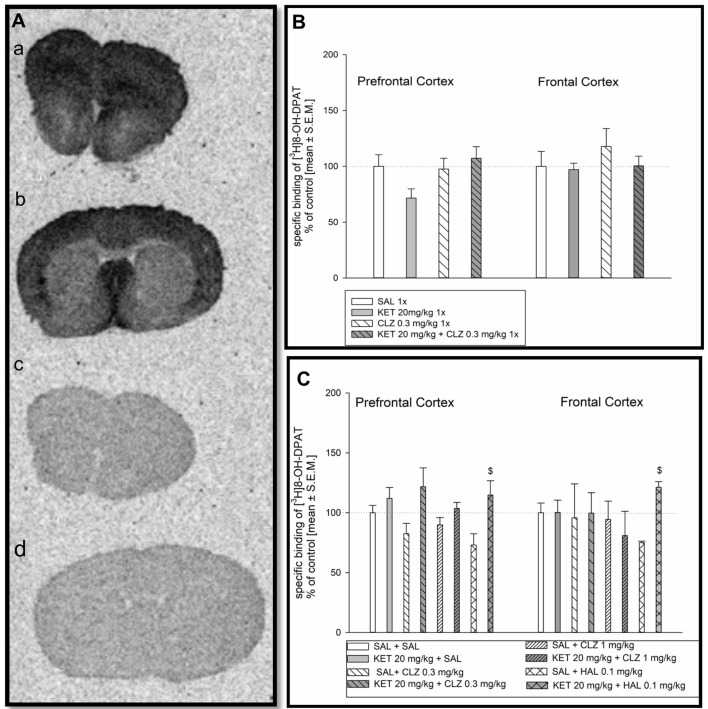
Effect of CLZ and HAL treatment on specific binding of [^3^H]8-OH-DPAT to 5HT_1A_R. **(A)** Representative autoradiograms of the total [^3^H] 8-OH-DPAT binding in mouse prefrontal cortex **(a)**; non-specific binding of [^3^H] 8-OH-DPAT in prefrontal cortex **(b)**; total [^3^H] 8-OH-DPAT binding in mouse frontal cortex **(c)**; non-specific binding of [^3^H]8-OH-DPAT in frontal cortex **(d)**. **(B)** Two-way ANOVA (SAL, KET) × (SAL, CLZ 0.3 mg/kg) did not show any significant differences in [^3^H] 8-OH-DPAT binding determined in the prefrontal and frontal cortex after acute CLZ treatment. **(C)** Repeated treatment by CLZ in two doses (0.3 mg/kg and 1 mg/kg) caused significant KET impact on [^3^H]8-OH-DPAT binding to 5-HT_1A_R in the prefrontal cortex (*F*_(1,27)_ = 6.381; *p* < 0.05), however, in the frontal cortex, no significant differences were observed. For sub-chronic HAL administration, two-way ANOVA analysis (SAL, KET) × (SAL, HAL) revealed also a significant effect of KET on the [^3^H]8-OH-DPAT binding in the prefrontal cortex and frontal cortex (*F*_(1,13)_ = 6, 609; *p* < 0.05 and *F*_(1,9)_ = 17.8; *p* < 0.01, respectively). The *post hoc* analysis revelead the statistical significant differences between KET+HAL 0.1 mg/kg vs. KET 20 mg/kg groups in frontal and prefrontal cortex, ^$^*p* < 0.05. The control data were standardized to 100%. The data represent the mean ± SEM.

Distribution of [^3^H]8-OH-DPAT binding to 5-HT_1A_R is presented in Figure 2A. Analysis of 5-HT_1A_ receptor density in mice receiving antipsychotic drugs in acute treatment paradigm did not indicate any significant differences in radioligand binding in prefrontal and frontal cortex (Figure 2B). In repeated administration, significant KET impact on [^3^H]8-OH-DPAT binding to 5-HT_1A_R in the prefrontal cortex was observed. Groups of mice receiving KET during first 7 days were characterized by higher density of 5-HT_1A_R in comparison to experimental group treated SAL for 7 days followed by replacement with antipsychotic drugs (Figure 2C). In frontal cortex, only sub-chronic treatment of KET + HAL 0.1 mg/kg induced a significant increase of [^3^H] 8-OH-DPAT binding to 5-HT_1A_ receptors in relation to SAL + HAL 0.1 mg group (Figure 2C).

### [^3^H]Ketanserin Binding to Serotonin 5-HT_2A_ Receptors and Analysis of Autoradiograms

**Figure 3:**
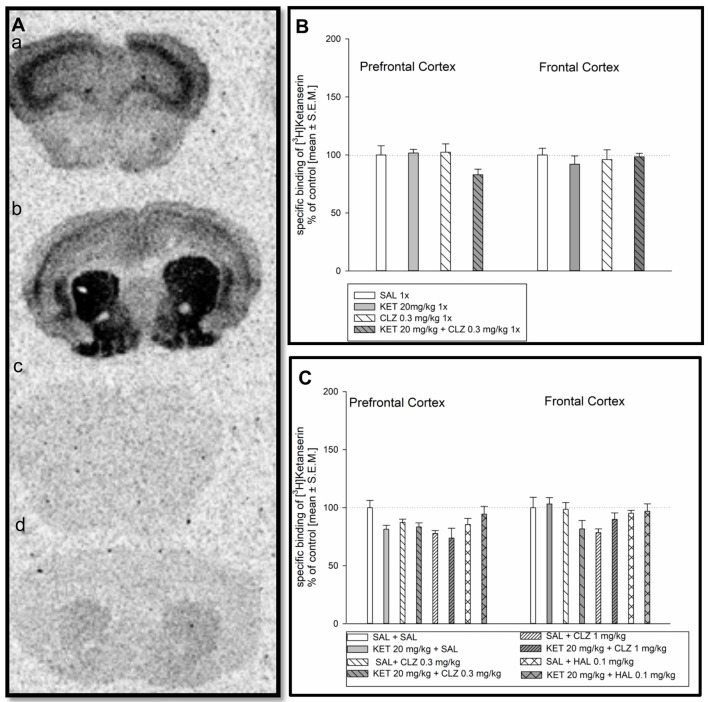
Effect of CLZ and HAL treatment on specific binding of [^3^H]ketanserin to 5HT_2A_R. **(A)** Representative autoradiograms of the total [^3^H]ketanserin binding in mouse prefrontal cortex **(a)**; non-specific binding of [^3^H]ketanserin in prefrontal cortex **(b)**; total [^3^H]ketanserin binding in mouse frontal cortex **(c)**; non-specific binding of [^3^H]ketanserin in frontal cortex** (d)**. **(B)** Two-way ANOVA (SAL, KET) × (SAL, CLZ 0.3 mg/kg) did not show any significant alterations in [^3^H]ketanserin binding determined in the prefrontal and frontal cortex. **(C)** For repeated administration, statistical analysis concerning prefrontal cortex did not indicate any significant drug’s effect on abundance of serotonin 5-HT_2A_ receptors. Two-way ANOVA (SAL, KET) × (SAL, CLZ 0.3 mg/kg, CLZ 1 mg/kg) and (SAL, KET) × (SAL, HAL) revealed a significant impact of CLZ (*F*_(2,22)_ = 4.290; *p* < 0.05), while the HAL treatment did not have any significant impact on the [^3^H]ketanserin binding in the brain frontal cortex. The control data were standardized to 100%. The data represent the mean ± SEM.

Representative autoradiograms of the distribution of [^3^H]ketanserin binding to serotonin 5-HT_2A_ receptors in the mouse prefrontal cortex and frontal cortex are showed in Figure 3A). Acute treatment of mice did not cause any significant changes in density of serotonin 5-HT_2A_ receptors in mouse prefrontal and frontal cortex (Figure 3B). Analysis of [^3^H]ketanserin binding to serotonin 5-HT_2A_ receptors in mouse frontal cortex after repeated drug administration releaved significant impact of CLZ 1 mg/kg treatment, while the HAL treatment did not have any significant impact (Figure 3C). Statistical analysis concerning prefrontal cortex did not indicate any significant drug’s effect on abundance of serotonin 5-HT_2A_ receptors (Figure 3C).

#### Co-localization of D_2_ Receptors and 5-HT_1A_ Receptors in the Mouse Brain Cortex

In order to determine whether D_2_ and 5-HT_1A_ receptors as well as 5-HT_1A_ and 5-HT_2A_ receptors can directly interact with each other, we studied the co-localization of the following pairs receptors: D_2_/5-HT_1A_ and 5-HT_1A_/5-HT_2A_ in mouse brain using immunohistochemistry and fluorescence microscopy.

**Figure 4:**
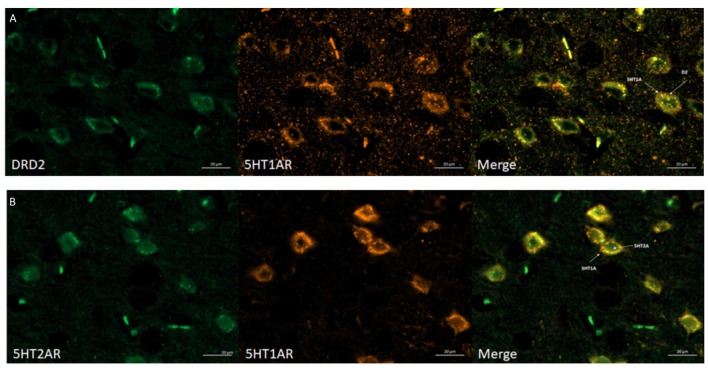
Immunohistochemical detection of co-localization of the 5HT_1A_R with D_2_R or 5HT_2A_R in mice frontal cortex.** (A)** Representative images of double-immunofluorescent staining for dopamine D_2_R (green) and serotonin 5HT_1A_R (orange) in the mouse cortex—merged (yellow). Scale bar: 20 μm. **(B)** Representative images of double-immunofluorescent staining for 5HT_2A_R (green) and serotonin 5HT_1A_R and (orange) in the mouse cortex—merged (yellow). Scale bar: 20 μm. White arrows indicated membrane localization and co-localization of studied receptors.

The obtained data indicated that dopamine D_2_ receptors co-localized with 5-HT_1A_ receptors and that 5-HT_1A_ co-localized with 5-HT_2A_ receptors in the mouse cortex (Figures 4A,B, respectively). All required positive and negative controls were done indicating that used antibodies are specific (data not shown). Antibodies were chosen based on literature (Ramírez et al., 2009; Noga et al., 2009; Yeung et al., 2010; Merlo et al., 2011; Hooper et al., 2016; Xiao et al., 2016). Moreover, we checked the specificity of antibodies by Western Blot, where different levels of receptors protein in different mouse brain structures were observed (data not shown).

### Control of PLA Technique

**Figure 5:**
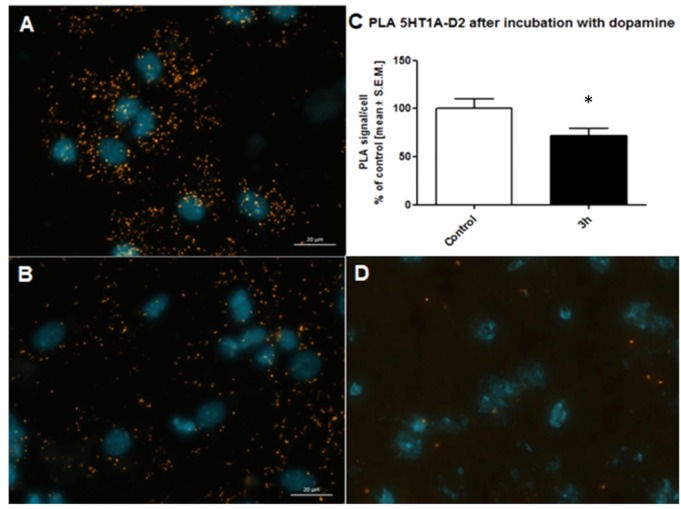
Control of specificity proximity ligation assay (PLA) assay. Representative fluorescence image of 5HT_1A_R-D_2_R PLA signal in mouse cortex neurons from control group **(A)** and neurons incubated with 10 μM dopamine for 3 h **(B)**. D_2_R internalization caused by dopamine decrease level of 5HT_1A_R-D_2_R interaction measured using PLA assay. The control data were standardized to 100%. The data represent the mean ± SEM and were analyzed using unpaired *t* test *p < 0.05 **(C)**. Fluorescence image showing lack of PLA signal which determine level of 5HT_1A_R-D_2_R interaction from corpus callosum indicating regional specificity PLA method **(D)**.

Antibody selection was preceded first in single recognition experiment follow by double recognition assay according to PLA method protocol (data not shown). To determine the method specificity, PLA was performed on mouse cortex neurons. Cells from the mouse cortex were isolated as described previously (Brewer and Torricelli, 2007). On day 7 of cells culture, cells were incubated with 10 μM dopamine for 3 h. Dopamine leads to internalization of D_2_ receptors (Bartlett et al., 2005) and helps determine the specificity of the PLA signal. As a result of the internalization of the dopamine D_2_ receptors at 10 μM concentration of dopamine, we observed a decrease of interaction between the 5-HT_1A_–D_2_ receptors (Figure 5C). Figures 5A,B showed fluorescence images in which difference in interaction between the 5-HT_1A_–D_2_ receptors in mouse visible as different level of PLA signal was observed. Moreover, to authenticate PLA method, we showed image from corpus callosum where PLA signal was not observed, which confirm specificity of PLA methods as well as antibodies (Figure 5D).

### Interaction of D_2_ and 5-HT_1A_ Receptors

The method of choice to study endogenous GPCR heteromers in the native tissue is the *in situ* PLA. The most popular format of this technique uses a pair of receptor-specific antibodies from different species, which are recognized by secondary antibodies with attached oligonucleotides. When the probes recognize the target, the attached oligonucleotides are then localized at a sufficiently close distance (less than 40 nm); proximity-dependent ligation forms a circular DNA template, which is thereafter amplified via rolling circle amplification. The product is visualized with a fluorescently labeled probe.

**Figure 6:**
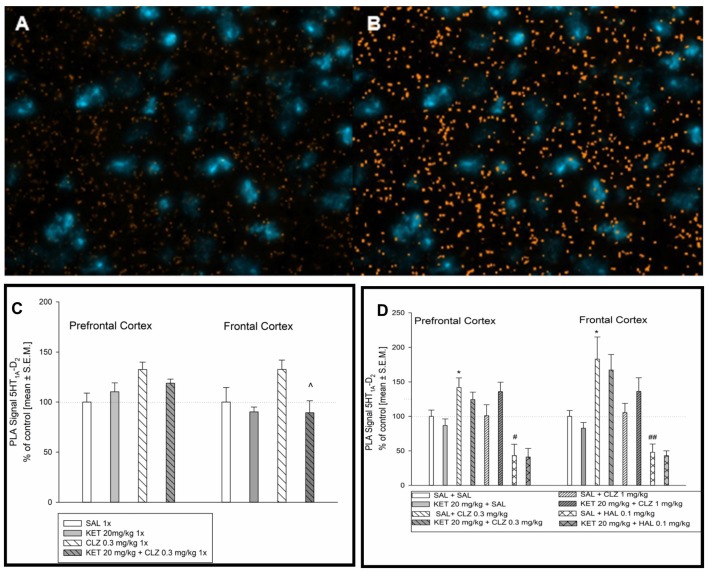
Effect of acute and sub-chronic antipsychotic drug’s treatment on the level of 5HT_1A_R-D_2_R interaction in mouse brain. Representative fluorescence image of D_2_R and 5HT_1A_R interaction after acute treatment **(A)**. Representative image after computer analysis of PLA signal 5HT_1A_R-D_2_R in mice frontal cortex **(B)**. For acute treatment experiments, two-way ANOVA (SAL, KET) × (SAL, CLZ 0.3 mg/kg) revealed significant impact of CLZ 0.3 mg on D_2_–5-HT_1A_ receptor in the prefrontal cortex (*F*_(1,14)_ = 6.564, *p* < 0.05) **(C)**. Statistical analysis (SAL, KET) × (SAL, CLZ 0.3 mg/kg) of PLA signals indicating D_2_–5-HT_1A_ interaction in the brain frontal cortex showed the influence of acute KET treatment (*F*_(1,14)_ = 6.634; *p* < 0.05). The *post hoc* analysis revelead the significant difference between KET+CLZ 0.3 mg/kg and CLZ 0.3 mg/kg group, ^^^*p* < 0.05. **(C)**. Analysis of sub-chronic treatment on D_2_–5-HT_1A_ receptor PLA signal (SAL, KET) × (SAL, CLZ 0.3 mg/kg, CLZ 1 mg/kg) and (SAL, KET) × (SAL, HAL) in a mouse brain tissue revealed significant effect of CLZ and HAL in the prefrontal cortex ((*F*_(2,22)_ = 4.630; *p* < 0.05) and (*F*_(1,14)_ = 14.97; *p* < 0.05), respectively) The *post hoc* analysis revealed statistical significant changes between CLZ 0.3 mg/kg vs. SAL group, *p < 0.05 and between HAL 0.1 mg/kg vs. SAL group, ^#^*p* < 0.05 **(D)**. Similairly, in mice frontal cortex, significant influence of CLZ and HAL sub-chronic administration on 5HT_1A_R-D_2_R interaction level was observed ((*F*_(2,19)_ = 9.755; *p* < 0.05) and (*F*_(1,13)_ = 24.73; *p* < 0.05), respectively). The *post hoc* analysis revealed statistical significant changes between CLZ 0.3 mg/kg vs. SAL group, *p < 0.05 and between HAL 0.1 mg/kg vs. SAL group, ^##^*p* < 0.01 **(D)**. The control data were standardized to 100%. The data represent the mean ± SEM.

Representative fluorescence images indicating D_2_ and 5-HT_1A_ receptor interaction is shown in Figure 6A. Figure 6B represents image after analysis done by microscope software assessing interaction level of D_2_R and 5-HT_1A_R in selected mouse brain regions. In prefrontal cortex, lower dose of CLZ (0.3 mg/kg) given acutely increased the level of D_2_–5-HT_1A_ receptor interaction in comparison to control group (Figure 6C). Analysis of PLA signal level in mouse frontal cortex indicated significantly lower D_2_–5-HT_1A_ receptor interaction in experimental groups: 1× KET and 1× KET + CLZ 0.3 mg (Figure 6C). Sub-chronic treatment of CLZ at 0.3 mg/kg increased the PLA signal but repeated administration of HAL led to decrease in D_2_–5-HT_1A_ receptor interaction, in comparison to control group in prefrontal cortex (Figure 6D). Similarly to the prefrontal cortex, CLZ 0.3 mg/kg administered sub-chronicaly increased the level of D_2_–5-HT_1A_ receptor interaction in the brain frontal cortex, in contrast to HAL which caused decrease in the interaction of this pair of receptors (Figure 6D).

### Interaction of 5-HT_1A_ and 5-HT_2A_ Receptors

**Figure 7:**
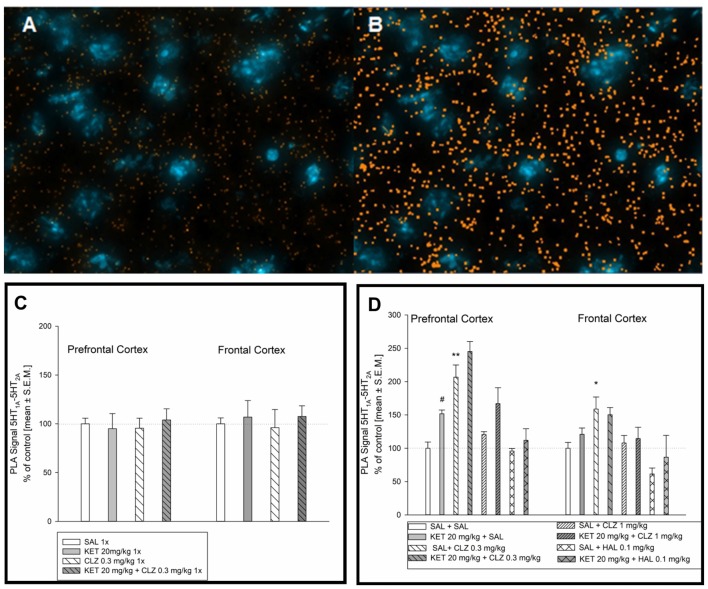
Effect of acute and sub-chronic antipsychotic drug’s treatment on the level of 5HT_1A_–5HT_2A_ interaction in mouse brain. Representative fluorescence image of D_2_R and 5HT_1A_R interaction after chronic treatment **(A)**. Representative image after computer analysis of PLA signal 5HT_1A_R-D_2_R in mice frontal cortex **(B)**. Two-way ANOVA analysis (SAL, KET) × (SAL, CLZ 0.3 mg/kg) did not show any significant alterations in 5HT_1A_–5HT_2A_ receptors interaction level in mice PFC and frontal cortex after acute treatment **(C)**. Statistical analysis of serotonin 5-HT_1A_–5-HT_2A_ receptor interaction (SAL, KET) × (SAL, CLZ 0.3 mg/kg, CLZ 1 mg/kg) in the prefrontal cortex after chronic CLZ treatment showed a significant effect of KET and CLZ (*F*_(2,25)_ = 7.534; *p* < 0.05; *F*_(1,25)_ = 14.08; *p* < 0.05; respectively). Concerning HAL treatment, two-way ANOVA (SAL, KET) × (SAL, HAL) revealed also a significant impact of KET (*F*_(1,12)_ = 5.953; *p* < 0.05). The *post hoc* analysis revealed significant differences between: KET 20 mg/kg vs. SAL group, ^#^*p* < 0.05 and between CLZ 0.3 mg/kg vs. SAL group, *p < 0.05, ^**^p < 0.01 **(D)**. In the brain frontal cortex, statistical analysis (SAL, KET) × (SAL, CLZ 0.3 mg/kg, CLZ 1 mg/kg) and (SAL, KET) × (SAL, HAL) revealed significant influence of chronic CLZ’s treatment on PLA signal level indicating serotonin 5-HT_1A_–5-HT_2A_ receptor interaction (*F*_(2,19)_ = 8.213; *p* < 0.05). The *post hoc* analysis revealed significant difference between CLZ 0.3 mg/kg and SAL group, *p* < 0.05 **(D)**. The control data were standardized to 100%. The data represent the mean ± SEM.

Figure 7A represents an image of serotonin 5-HT_1A_–5-HT_2A_ interaction in the mouse brain. Figure 7B shows image after computer analysis of determined PLA signal level. In brain of mouse treated in acute paradigm, any significant alterations in receptors interaction level was not observed (Figure 7C). Analysis of serotonin 5-HT_1A_–5-HT_2A_ receptor interaction in the PFC after chronic treatment showed a significant effect of KET and CLZ 0.3 mg/kg. In all KET receiving groups as well as in SAL + CLZ 0.3 mg/kg group, an increase in PLA signal for 5-HT_1A_–5-HT_2A_ receptors was observed in the PFC, in comparison to control group (Figure 7D). In the brain frontal cortex, repeated treatment SAL + CLZ 0.3 mg/kg induced increase in serotonin 5-HT_1A_–5-HT_2A_ receptor interaction (Figure 7D).

## Discussion

The main finding of this manuscript is the demonstration that the GPCR interaction can be important in the action of KET and CLZ.

It seems especially important in the field of antipsychotic drugs, which are effective in the clinic but induce adverse side effects. One such drug is CLZ, which shows a wide receptors profile. CLZ is classified as atypical neuroleptic because of affinity to serotonin receptors; its affinity for 5-HT_2A_R is greater than for D_2_R (Meltzer, 1999). It has been shown that CLZ has a high *in vitro* affinity for 5-HT_2A_ receptor and behaves as partial agonist *in vivo* at 5-HT_1A_R (Kargieman et al., 2012). With their moderate *in vitro* affinity for 5-HT_1A_, CLZ and other atypical antipsychotic drugs act as agonists at 5-HT_1A_ receptor *in vivo* to increase PFC dopamine release (Rollema et al., 1997; Ichikawa et al., 2001; Díaz-Mataix et al., 2005; Bortolozzi et al., 2010; Purkayastha et al., 2012). PFC plays a fundamental role in higher brain functions and numerous observations suggest an abnormal function of this cortical area in schizophrenia (Goldman-Rakic, 1994; Knable and Weinberger, 1997; Edwards et al., 2010). Moreover, the non-competitive NMDA receptor antagonists, such as phencyclidine (PCP) or KET exacerbate clinical symptoms in patients with schizophrenia and induce behavioral alterations that resemble schizophrenia symptoms in healthy individuals and experimental animals (Kargieman et al., 2012). In our previously behavioral studies using ASST test in mouse (Szlachta et al., 2017), we have demonstrated that CLZ 0.3 mg/kg reverses the KET activity in the crucial phase of this test. This effect was interpreted via the possible specific action of this substance on serotonin and dopamine heteromers. Because of this, the goal of our work was to demonstrate that the molecular mechanism of the unique action of CLZ is dependent on receptor heteromers. The present study was performed using PLA technique, which enables to monitor the dimerization of a given pair of receptors in the mouse brain. In our previous study we observed in locomotor activity test, that CLZ 1 mg/kg after acute treatment induced sedation effects. Therefore, in this study, we also tested only low-dose CLZ in acute paradigm treatment (Szlachta et al., 2017).

The PLA experiments were preceded by the receptor autoradiography (analysis on the same brain samples) in order to determine whether the treatment of CLZ, HAL, or KET influenced the levels of the studied receptors in the PFC and frontal cortex.

The interaction between receptors is possible only when they co-localize therefore we first confirmed that indeed, 5-HT_1A_ and dopamine D_2_ receptors co-localize in the mouse PFC and frontal cortex. The co-localization of 5-HT_1A_ and D_2_ receptors was also observed in the mouse PFC by Łukasiewicz et al. (2016).

In our study, we observed 5-HT_1A_–D_2_ physical interaction in PFC and frontal cortex in mice. There is increasing interest in antipsychotics intended to manage positive symptoms via D_2_ receptor blockade and improve negative symptoms and cognitive deficits via 5-HT_1A_ receptor activation (Newman-Tancredi and Kleven, 2011). In this context, we studied if antipsychotic drugs affected the physical interaction of these receptors. The functional consequences of the signaling pathways mediated by 5-HT_1A_–D_2_ interaction are still not known in detail. Łukasiewicz et al. (2016) indicated that upon incubation of cells with antipsychotic drugs, different second messenger pathways are activated depending on whether D_2_ and 5-HT_1A_ receptors are expressed alone or together (Łukasiewicz et al., 2016). It is interesting that in our studies KET did not influence the 5-HT_1A_–D_2_ interaction, whereas CLZ in low-dose (0.3 mg/kg) increased the interaction of this pair of receptors in the mouse brain. The impact of CLZ on the 5-HT_1A_–D_2_ heteromers confirms the results obtained in *in vitro* study by Łukasiewicz et al. (2016). However, the scope of the present research did not cover the functional significance of this phenomenon, but it will certainly be a subject of further studies. Interestingly, on autoradiographs, we did not observe any changes in receptor density following KET or CLZ treatment. On the other hand, administration of HAL significantly decreased the density of dopamine D_2_R, so the decreased PLA signal indicating the decreased receptor interaction observed after HAL treatment might have resulted from the antagonist action of this drugs on D_2_R. However, it is interesting that in receptor autoradiography studies we observed the lower level of 5-HT_1A_R following HAL administration and this effect was reversed by KET pretreatment. Since the physical interaction between 5-HT_1A_ and NMDA receptors in the PFC has been reported (Yuen et al., 2005) and HAL pretreatment reduces impairments in cognitive function produced by KET (Krystal et al., 1999) the mechanism of this interaction can be the results of receptor dimerization. In our study, we treated mice first with KET, and then with HAL. Hence, it is tempting to suggest that earlier administration of KET might have induced conformational changes in the binding site of the 5-HT_1A_R.

Recently, using PLA methodology, has been evidence for the existence of brain 5-HT_1A_–5-HT_2A_ isoreceptor complexes validated in cellular models with bioluminescence resonance energy transfer (BRET2) assay. The authors demonstrated the existence of 5-HT_1A_–5-HT_2A_ isoreceptor complexes in the dorsal hippocampus and the anterior cingulate cortex (Borroto-Escuela et al., 2017a). Both 5-HT_1A_R and 5-HT_2A_R are abundantly expressed in rodent PFC (Pompeiano et al., 1992, 1994; Santana et al., 2004), where they are mostly co-expressed (Amargós-Bosch et al., 2004). They mediate opposing actions: 5-HT_1A_R is inhibitory, while 5-HT_2A_R is excitatory of 5-HT and selective agonists (Araneda and Andrade, 1991; Marek and Aghajanian, 1999; Amargós-Bosch et al., 2004; Puig et al., 2005). It has been shown that 5-HT_1A_R and 5-HT_2A_R receptors mediate the changes in cortical dopaminergic transmission induced by atypical antipsychotic drug. Atypical neuroleptics via blockade of 5-HT_2A_R and D_2_R, may promote the ability of 5-HT_1A_R stimulation to increase PFC dopamine release (Ichikawa et al., 2001; Celada et al., 2004). Therefore, we also studied this pair of receptors in the context of potential dimerization. First, we found the co-localization of these receptors in the mouse brain. We observed the co-localization of 5-HT_1A_R and 5-HT_2A_R in the mouse prefrontal cortex and frontal cortex. To the best of our knowledge this is the first time observation in mice and it is complementary to observation in rats by Wędzony et al. (2008). We were also able to observe the interaction between 5-HT_1A_R and 5-HT_2A_R in the studied brain regions. The observation of interaction between HT_1A_–5-HT_2A_ it can be confirmed by results obtained by Borroto-Escuela et al. (2017a). Intriguing finding in current work is that KET had impact on the interaction of this pair of receptors, while it did not affect the 5-HT_1A_–D_2_ receptor interactions. This result was also independent of any changes in receptors density (no changes in receptor autoradiography using [^3^H]ketanserin and [^3^H]8-OH-DPAT). However, it has been shown that KET exhibited a high affinity only for the 5-HT_2A_R and showed a much lower affinity for the low affinity state of this receptor (Kapur and Seeman, 2002). In addition, we have shown the effect of low-dose CLZ on 5-HT_1A_–5-HT_2A_ receptor interaction. KET and CLZ induced a synergistic effect, whereas higher-dose CLZ had no effect. This difference is puzzling; however, such phenomenon has been already observed in our previous study on uncoupled dopamine D_1_–D_2_ receptor heterodimers (Faron-Górecka et al., 2008). The results obtained in the present study indicate that KET had an impact on 5-HT_1A_–5-HT_2A_ receptor interaction, but they do not explain the behavioral outcomes obtained in ASST. If the effect of CLZ in KET-induced ASST test in mice was based on a specific action of this receptor pairs, we should observe different (opposing) effect of KET and CLZ on the formation of the studied pair of GPCR.

The results obtained by us in biochemical studies point to the selective action of individual KET, CLZ, or HAL drugs on the potential formation of heteromers, which may have impact on further investigation not only of the unique action of CLZ but also of KET, an important drug not only in schizophrenia but also in depression. However, we have to be aware, that presented results demonstrate only antipsychotic impact on receptors interaction but further research is required to prove functional dimerization of GPCR.

### Limitation of Studies

The best control of PLA methods specifity would be experiments using knock-out mice. This approach let to assess what is the level of PLA signal background without one of receptor and then subtraction this background from experimental group. Presented data lack such a control. But our studies include the appropraite controls which in our opinion are sufficient to state that PLA assay is reliable: (1) *in vitro* experiment with 10 μM dopamine, inducing dopamine D_2_ receptors internalization and PLA signal is decreased; (2) according to literature, in corpus callosum, dopamine D_2_ receptors do not exist and we did not observe any D_2_R-5HT_1A_R PLA signal what proves method and antibody specificity; and (3) single recognition experiment (PLA method used to assess expression level of one protein) indicated the level of D_2_ receptors in the striatum twice as high as in the prefrontal cortex.

Many control experiments also have been done which confirmed antibodies specificity: (1) D_2_ receptors were not expressed in all brain regions, i.e., D_2_ receptors expression was observed in the brain cortex but was not observed in the hippocampus; similarly, D_2_ receptors were not expressed in all cortex layers but only in particular ones; (2) cellular localization of D_2_ receptors and 5HT_1A_ receptors determined by our antibodies are different and signals from two antibodies were not overlapped in all cells; not every cell which expressed D_2_ receptor also had 5HT_1A_ receptor; and (3) images from single recognition PLA experiment not show any background what proves specificity of our antibody because according to Duolink manufacturer’s protocol, when unspecific antibody is used, high background is visibled, due to the sensitivity of the assay.

There is a discussion on the interpretation of results using available techniques to observe the GPCRs interaction. It has been shown that this phenomenom could activate the new signaling pathways or lead to functional crosstalk (Albizu et al., 2011; Ferré et al., 2014; Borroto-Escuela et al., 2016). Moreover it has been raised that the GPCRs interaction studied *in vitro* by FRET methods as well as in native tissue by PLA assay determine only close distance between them (in case of PLA method it is 17 Å), so these methods do not provide sufficient evidence to talk about their direct physical interaction and this is imprecise term. Due to currently available methods, published articles regarding GPCRs heterodimerization indicate that their interaction could lead to molecular crosstalk between receptors and cause functional consequence rather than their physical contact heterodimerization (Bouvier and Hébert, 2014; Lambert and Javitch, 2014). GPCR dimerization is still controversial, although many publications indicate that research using these techniques they are evidence proof of GPCRs dimerization (Gomes et al., 2016; Rico et al., 2017).

## Author Contributions

AF-G, MS and MD-W designed the study and wrote the protocol. MS and AF-G performed experiments using the PLA technique. MKuśmider performed autoradiography experiments. PP and JS administered drugs and performed tissue extraction for biochemical analysis. DŻ and MKolasa performed technical controls for PLA methods. AF-G and MS wrote the first draft of the manuscript. All authors contributed to and have approved the final manuscript.

## Conflict of Interest Statement

The authors declare that the research was conducted in the absence of any commercial or financial relationships that could be construed as a potential conflict of interest.
